# Gastric Cancer in Young Patients

**DOI:** 10.1155/2013/981654

**Published:** 2013-12-07

**Authors:** Manzoor A. Dhobi, Khursheed Alam Wani, Fazl Qadir Parray, Rouf A. Wani, Mohd Lateef Wani, G. Q. Peer, Safiya Abdullah, Imtiyaz A. Wani, Muneer A. Wani, Mubashir A. Shah, Natasha Thakur

**Affiliations:** Department of General and Minimal Invasive Surgery, Sher-i-Kashmir Institute of Medical Sciences, Soura, Srinagar 190011, India

## Abstract

*Aim.* The aim of this study was to see the clinical, pathological, and demographic profile of young patients with stomach carcinoma besides association with p53. *Patients and Methods.* Prospective study of young patients with stomach carcinoma from January 2005 to December 2009. A total of 50 patients with age less than 40 years were studied. *Results.* Male female ratio was 1 : 1.08 in young patients and 2.5 : 1 in older patients. A positive family history of stomach cancer in the first degree relatives was present in 10% of young patients. Resection was possible only in 50% young patients. 26% young patients underwent only palliative gastrojejunostomy. The most common operation was lower partial gastrectomy in 68%. Amongst the intraoperative findings peritoneal metastasis was seen in 17.4% in young patients. 50% young patients presented in stage IV as per AJCC classification (*P* value .004; sig.). None of the patients presented as stage 1 disease in young group. *Conclusion.* Early detection of stomach carcinoma is very important in all patients but in young patients it is of paramount importance.

## 1. Introduction

Gastric cancer is the second leading cause of death due to malignancy worldwide and occurs most frequently in the age group of 50–70 years [1–3]. However, over the past half century several studies have reported on the clinical and pathological features of gastric carcinoma in young adults in the range of 2%–8% in different series [4].

The incidence of gastric cancer is the highest in Japan, China, south America and eastern Europe and the lowest in the United States [2]. Gastric cancer is the third most common cancer in Kashmir only superseded by esophageal and lung cancer [5].

Considerable evidence suggests the role of genetic factors in the pathogenesis of gastric carcinoma. Clustering of this disease within families has been reported in Bonaparte's family. Napoleon, his father, his grand father, and several of his siblings died of cancer stomach [2]. Inherited or familial gastric cancer and hereditary diffuse gastric cancer (HDGC) are common in patients younger than 40 years of age. Patients with hereditary nonpolyposis colorectal cancer (Lynch syndrome II) are at increased risk of stomach cancer. First degree relatives of patients with gastric cancer have a two- to threefold increased risk of developing this disease [6]. There is an increased risk of gastric cancer in people with blood group A [2].

Diets rich in salted, smoked, or poorly preserved foods are associated with increased risk of cancer stomach, whereas diets rich in fruits and vegetables are associated with decreased risk. Foods rich in nitrates, nitrites, and secondary amines can combine with N-nitro compounds which induce gastric tumors in animals [6]. Smokers have 1.5- to 3.0- fold increased risk of cancer stomach. Alcoholics have also an increased risk of developing this disease [2].

A near universal finding in young patients has been the high frequency of advanced lesions and undifferentiated tumors at presentation in comparison with older patients; this has often been attributed to the delay in diagnosis [7]. Gastric cancer in the young patients spreads more rapidly and is biologically more aggressive [8]. Young patients less likely present as gastroesophageal junction growth as compared to antral growth [9].

Our valley falls in the high endemic zone of stomach cancer. It is the third most common cancer in valley. Most patients are older than 50 years. However, sometimes we do come across the patients with stomach cancer in their third or fourth decade of life. This motivate us to undertake this study of stomach cancer in young patients to see their demographic and clinicopathological profile and their association with p53 gene.

## 2. Methods

The present study was a prospective conducted in the Department of General Surgery and Department of Immunology and Molecular Medicine, Sher-i-Kashmir Institute of Medical Sciences, Srinagar, from January 2005 to December 2009. Young patients were defined as less than 40 years of age.

A detailed history, general physical exam, and routine investigations were carried out. Every patient underwent abdominal ultrasonography and contrast enhanced computerized tomogram (CECT) for proper preoperative staging. Patients were optimized for any comorbid condition. Fine needle aspiration cytology (FNAC) of any extra abdominal enlarged lymph node was carried out to rule out metastasis. All the patients who after clinical and radiological assessment had an operable tumor were subjected to laparotomy for any possible resective or bypass procedure. Histological examination of resected specimen was conducted to know the type, grade, and stage of tumor. Specimens from 7 young and 16 old patients were taken from normal tissue, tumor tissue, and blood and lymph nodes and were sent to the department of immunology and molecular medicine for the study of p53. DNA extraction was carried by using a phenol-chloroform method. PCR amplification technique was standardized to amplify 2nd para EXON 5, 6, 7, and 8 of p53 gene from genomic DNA. Mutation in the amplified exons of p53 was asserted by a single stranded conformational polymorphism (SSCP) and restriction fragment length polymorphism. Samples showing SSCP shift migration were sequenced to identify the type of mutations. All the cases were discharged after stabilization, followed, and regularly monitored for any complication. Adjuvant treatment was used in most of the patients. Finally, whole data was compiled and analyzed statistically. Data was described in percentages and chi-square, and odds ratio analysis was used for valid inferences. Software, microsoft excel, Minitab, and SPSS (11.5 versions) were used for statistical analysis.

## 3. Results

**Figure 1:**
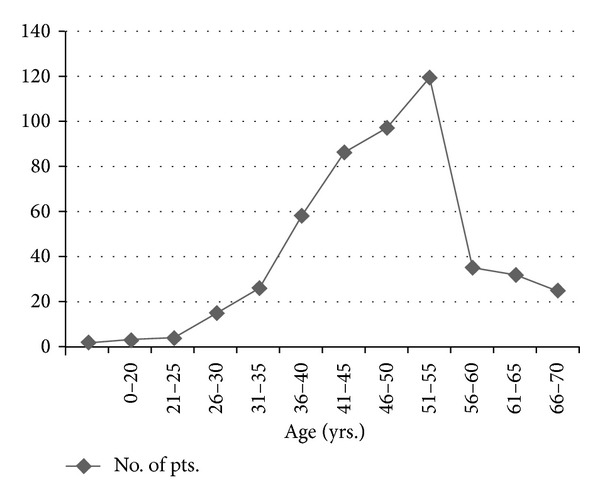


Analysis of 502 patients of stomach cancer admitted in the study period was done. Out of these studied patients 50 patients belonged to less than or equivalent to 40 years of age group (Figure 1). Around 10% of patients were younger than 40 years. Male female ratio was 1 : 1.08 in young and 2.5 : 1 in older patients. A positive family history of stomach cancer in the first degree relatives was present in 10% of young and 3% of old patients which was statistically significant (*P* value 0.006). 44% of patients from young age group had history of smoking whereas 62% from old age group had the same history (*P* value 0.014 sig). Main comparative symptomatology between the 2 groups was weight loss 64% in <40 yrs and 80% in >40 yrs age groups, anorexia 52% versus 79% (0.000; sig), malena 16% versus 6%, constipation 4% versus 32%, and dysphagia 2% versus 22%; all these findings were statistically significant, whereas rest of the symptoms on comparison were statistically insignificant. Amongst the signs, gastric splash (succussion splash) 145 versus 35% had a statistically significant *P* value (0.003), whereas rest of the signs were not significant statistically. The most common type of lesion in young patients was infiltrative type (38%) while it was polypoid (58%) in older patients and this difference again was statistically significant as the distribution of lesion in various parts of stomach was statistically significant. 71% of young patients had poorly differentiated lesions and 40% older patients had moderately differentiated lesions. 69% young and 33% old patients had diffuse type of lesions, whereas 25% young and 58% old patients had intestinal type of lesions. Resection was possible in 50% young versus 69% old patients. 26% young versus 10% old patients underwent only palliative gastrojejunostomy. The most common operation was lower partial gastrectomy in 68% versus 49%. Amongst the intraoperative findings peritoneal metastasis was seen in 17.4% in young patients versus 6.5% in old patients. The rest of all findings on comparison were statistically insignificant. 50% young versus 35% old patients presented in stage IV as per AJCC classification (*P* value 0.004; Sig). None of the patients presented as stage 1 disease in young group. Chemoradiotherapy for young versus old patients was 66.5% versus 51%; only chemotherapy was 27.5 versus 42%; and only radiotherapy was 6% versus 7% given as adjuvant therapy. Most common complication of surgery was wound infection 9% versus 14% followed by ileus 10% versus 11%. The survival of young patients is shown in Table 1. Mean survival was 10.3 months. Out of 33 young patients in prospective study, 16 died during the study period. Genetic alterations of p53 gene in gastric cancer are shown in Tables 2(a), 2(b), and 2(c).

## 4. Discussion

The incidence of gastric cancer is the highest in Japan, China, south America and eastern Europe and the lowest in the United States [2]. Gastric cancer is the third most common cancer in Kashmir only superseded by esophageal and lung cancer. The incidence of stomach cancer in young adults in our series was comparable to others [10, 11]. However, some authors reported incidence which was lower than what we observed [5]. The apparent increases in the recent few decades may be due to the fact that people are now better educated, more health conscious, and economically better off to seek the medical advice at any earlier stage. Male to female sex ratio was 1 : 1.08 amongst young patients and 2.5 : 1 in older patients which corresponds with what was reported by other authors [3, 12, 13]. The cause of higher frequency in young women is unknown. The reason for male preponderance in older patients could be due to more frequent and longer exposure to the environmental carcinogens. The age distribution in our study corresponds with other authors [3, 10]. Family history of stomach cancer in young patients was statistically significant in our study group (10% versus 3%, *P* = 0.006) which coincides with other studies [3], but lesser than what was reported by others [14, 15], that is, 17%–25%. One of the reasons of lower incidence of family history in our patients may be due to lower rate of literacy than that of Japan and most of the patients might not be able to recall the disease of their relatives.

The mean duration of symptoms in our series was 5.7 ± 5.0 months, corresponding with the finding of other authors [16]. As observed by other authors polypoid growth was more common than infiltrative growth in our series [13]. Poorly differentiated carcinoma is more common in young patients as reported by many authors [14]. The prognosis of gastric cancer decreases with increase in the grade of the lesion. The higher the grade, the worse the prognosis [11, 13]. Resection was possible only in 50% of the young and in 68.6% of old patients, procedures were done as per the indication, which corresponds with other studies [12]. The international comparison reveals that resection rate for gastric cancer is significantly more in Japan than the rest of the world; even our resection rate is far lower than what was reported by Japanese in the literature [16] (100% resectability rate); the reason is early detection of cancers.

Majority of our patients had advanced disease at presentation. 50% of the young and 35% of old patients were in stage IV. We had only 9% of patients from older group in stage I. Identical observations were made by other authors [12].

Our wound infection rate and rate of anastomotic leak were similar to others [1].

Seven out of 30 (23.3%) young and 16 out of 30 (53.3%) old patients had harbored mutations in p53. All the mutations identified were sporadic. No change was found in p53 gene derived from blood or normal tissues of the same patient. In all 23 tumors, a total of 23 mutations (5 insertions, 17 single base, 1 Para5 Tandem… double base substitution) in p53 were detected, all of which were somatic in nature. Mutation pattern data of p53 revealed a high percentage of insertions (6/23, 26%), G:C>A:T (at CpG site); (4/23, 17.4%), A:T>G:C, (8/23, 34.8%) transition mutations and G:C>C:G (5/23, 21.8%) transversions respectively. Analysis of the mutation spectrum revealed a number of salient and interesting features which included high frequency of A:T to G:C substitutions. The presence of p53 mutations in sporadic gastric cancer patients showed some statistically significant association with older patients (*P* = 0.034; OR = 3.8) as compared to young patients [17]. P53 mutations were significantly more frequent in intestinal type than those in diffuse type of tumors in both groups (*P* = 0.035; OR = 3.68). Our findings are consistent with Tolbert et al. [18] and Hsieh et al. [19], where p53 mutations were also frequent in smokers as compared to nonsmokers (*P* = 0.003; OR = 6.07).

The mean survival of our young patients was 10.3 months. Sixteen of our young patients died during the study period. Four patients with stage II disease showed a mean survival of 17.2 months and amongst them 2 patients after chemotherapy showed a mean survival of 18.6 months. These observations correspond to other studies [13]. In young patients the main cause of death was advanced disease and in older patients it was due to associated comorbid conditions.

The overall lower survival rate is like from rest of the world, but less than Japan. It can be attributed to the fact that percentage of early gastric cancer patients in our study was very low and can also be attributed to delay in diagnosis and high percentage of poorly differentiated lesions in young patients.

## 5. Conclusion

Thus, we conclude our study with an emphasis that early detection of carcinoma stomach is very important in all patients but in young patients it is of paramount importance. The said entity is, no more, a disease of the old people only, and time and again vague prescriptions of H2 blockers and proton pump inhibitors for dyspeptic symptoms even in young patients might be just a denial to rule out the possibilities of an early lesion and of making it progress to a stage where all medical armamentarium is rendered helpless.

## Figures and Tables

**Table 1:** Stage wise survival of studied subjects (young patients).

Stages	Adjuvant therapy	No. of patients	Survival (months)
Stage II	Yes	2	18.6
	No	2	16.6
Stage IIIA	Yes	4	14.2
	No	1	7.5
Stage IIIB	Yes	5	12.5
	No	2	8.5
Stage IV	Yes	2	7.85
	No	15	7

**Table:** (a)

Gender	<40 yrs		>40 yrs		Results
	*N*	%	*N*	%	
Male	4	25	12	57	
Female	3	21	4	44.4	

Total	7	23.3	16	53.3	OR = 3.8, *P* = 0.034 (sig)

**Table:** (b)

Smoking	<40 yrs		>40 yrs		Results
	*N*	%	*N*	%	
Yes	5	38.5	14	77.7	
No	2	11.8	4	33.3	

Total	7	23.3	16	53.3	OR = 6.07; *P* = 003

**Table:** (c)

Histopathology	<40 yrs		>40 yrs		Results
	*N*	%	*N*	%	
Diffuse type	4	18	5	21.7	
Intestinal type	3	37.5	11	64.7	

Total	7	23.3	16	53.3	OR = 3.68, *P* = 0.035

## References

[1] Llanos O, Butte JM, Crovari F, Duarte I, Guzmán S (2006). Survival of young patients after gastrectomy for gastric cancer. *World Journal of Surgery*.

[2] Fuchs CS, Mayer RJ (1995). Medical progress: gastric carcinoma. *New England Journal of Medicine*.

[3] Theuer CP, De Virgilio C, Keese G (1996). Gastric adenocarcinoma in patients 40 years of age or younger. *American Journal of Surgery*.

[4] Levine MS, Laufer I, Thompson JJ (1983). Carcinoma of the gastric cardia in young people. *American Journal of Roentgenology*.

[5] Rasool MT, Lone MM, Wani ML, Afroz F, Zaffar S, Mohib-ul Haq M (2012). Cancer in Kashmir, India: burden and pattern of disease. *Journal of Cancer Research and Therapeutics*.

[6] Mercer DW, Robinson EK *Stomach. Sabiston's Text Book of Surgery*.

[7] Bellegie NJ, David C, Dahlin DC (1953). Malignant disease of the stomach in young adults. *Annals of Surgery*.

[8] Hall TJ, Moulder J, Hsu HS, Achord J, Scott-Conner CE (1993). Gastric carcinoma among younger individuals in Mississippi. *Southern Medical Journal*.

[9] Theuer CP, Kurosaki T, Taylor TH (1998). Unique features of gastric carcinoma in the young. *Cancer*.

[10] Katai H, Sasako M, Sano T, Maruyama K (1996). Gastric carcinoma in young adults. *Japanese Journal of Clinical Oncology*.

[11] Santoro R, Carboni F, Lepiane P, Ettorre GM, Santoro E (2007). Clinicopathological features and prognosis of gastric cancer in young European adults. *British Journal of Surgery*.

[12] Tso PL, Bringaze WL, Dauterive AH (1987). Gastric carcinoma in the young. *Cancer*.

[13] Matley PJ, Dent DM, Madden MV, Price SK (1988). Gastric carcinoma in young adults. *Annals of Surgery*.

[14] Ramos-Dela Medina A, Salgado-Nesme N, Torres-Villalobos G, Medina-Franco H (2004). Clinicopathologic characteristics of gastric cancer in a young patient population. *Journal of Gastrointestinal Surgery*.

[15] Umeyama K, Sowa M, Kamino K (1982). Gastric carcinoma in young adults in Japan. *Anticancer Research*.

[16] Mori M, Sugimachi K, Ohiwa T (1985). Early gastric carcinoma in Japanese patients under 30 years of age. *British Journal of Surgery*.

[17] Rugge M, Arslan P, Egarter-Vigl E (2000). The p53 gene in patients under the age of 40 with gastric cancer: mutation rates are low but are associated with a cardiac location. *Journal of Clinical Pathology*.

[18] Tolbert D, Fenoglio-Preiser C, Noffsinger A (1999). The relation of p53 gene mutations to gastric cancer subsite and phenotype. *Cancer Causes and Control*.

[19] Hsieh U, Hsieh JT, Wang LY, Fang CY, Chang SH, Chen T (1996). p53 mutations in gastric cancers from Taiwan. *Cancer Letters*.

